# Associations of Fish and Omega-3 Fatty Acids Consumption With the Risk of Venous Thromboembolism. A Meta-Analysis of Prospective Cohort Studies

**DOI:** 10.3389/fnut.2020.614784

**Published:** 2020-12-17

**Authors:** Yi Zhang, Jun Ding, Hongbin Guo, Jieyu Liang, Yusheng Li

**Affiliations:** ^1^Department of Orthopaedics, Xiangya Hospital, Central South University, Changsha, China; ^2^Changsha Social Work College, Changsha, China

**Keywords:** fish consumption, omega-3 fatty acids consumption, venous thromboembolism, meta-analysis, prospective cohort studies

## Abstract

**Objective:** This study aims to investigate the effect of fish and omega-3 fatty acids consumption on the risk of VTE.

**Methods:** A comprehensive literature search in the databases of PubMed, Web of Science, and Embase (up to September 2020), was conducted to identify the prospective cohort studies concerning the associations of fish and omega-3 fatty acids consumption with the risk of VTE. The pooled relative risk (RR) of VTE for the highest vs. lowest category of fish and omega-3 fatty acids consumption, as well as their corresponding 95% confidence interval (CI) were calculated.

**Results:** A total of seven articles with eight prospective cohort studies were included. Specifically, six studies were related to fish consumption, and the overall multi-variable adjusted RR showed no significant relationship between fish consumption and the risk of VTE (RR = 1.02, 95% CI: 0.93–1.11; *P* = 0.709). In the four studies related to omega-3 fatty acids consumption, the overall multi-variable adjusted RR suggested that omega-3 fatty acids consumption was associated with a lower risk of VTE (RR = 0.89, 95% CI: 0.80–0.98; *P* = 0.024). Moreover, two studies were related to recurrent VTE, and the overall multi-variable adjusted RR demonstrated a significant inverse association between omega-3 fatty acids consumption and the risk of recurrent VTE (RR = 0.45, 95% CI: 0.25–0.81; *P* = 0.008).

**Conclusion:** Although current evidence is still insufficient to demonstrate any relationship between fish consumption and the risk of VTE, omega-3 fatty acids consumption seems to be associated with a lower risk of both VTE and recurrent VTE. Further large well-designed prospective cohort studies are warranted to elaborate the issues examined in this study.

## Introduction

Venous thromboembolism (VTE), encompassing deep vein thrombosis (DVT) and pulmonary embolism (PE), is a common cardiovascular disease (CVD) in adult populations (1–3). As a major cause of cardiovascular mortality, VTE constitutes a significant public health burden due to debilitating long-term complications (4, 5). In contrast with the declining rate of arterial CVD over the past decades (6, 7), the incidence of VTE remains stable or even slightly increased (8, 9). Moreover, VTE is also considered as a chronic and recurrent disease. Although anticoagulant therapy efficiently prevents VTE recurrence, approximately one-third of patients suffer recurrent VTE within 10 years (10, 11). Furthermore, the risk of mortality in patients with VTE is higher than that in general population (12). Thus, there is an urgent need to reduce the VTE risk. The identification of lifestyle factors influencing VTE appears to be an important step in both primary and secondary prevention.

Fish serves as an important determinant of healthy diet, and omega-3 fatty acids (eicosapentaenoic acid [EPA], docosapentaenoic acid [DPA], and docosahexaenoic acid [DHA]) are identified as key bioactive compounds (13). Omega-3 fatty acids are reported to be associated with downregulation of inflammation (14), platelet function (15), platelet-endothelium interactions (16, 17), and tissue factor expression (18), which are key pathways in VTE pathogenesis. To our best knowledge, the effect of fish and omega-3 fatty acids consumption on the risk of VTE has been investigated by a number of prospective cohort studies with conflicting results (13, 19–24). In 2016, a systematic review also indicated that the epidemiological evidence is insufficient to demonstrate any relationship between fish consumption and risk of VTE (25). However, the estimate effects were not pooled and omega-3 fatty acids and recurrent VTE were also ignored. To address these issues above, a meta-analysis of prospective cohort studies was therefore systematically performed. It was hypothesized that fish and omega-3 fatty acids consumption was associated with a lower risk of VTE.

## Methods

### Search Strategy

This meta-analysis was performed according to the Preferred Reporting Items for Systematic review and Meta-analyses (PRISMA) guidelines (26). The electronic databases of PubMed, Web of Science, and Embase were searched up to September 2020, using a series of logic combinations of keywords and in-text words that are related to venous thromboembolism (“venous thromboembolism,” “deep vein thrombosis,” “pulmonary embolism”), fish (“fish,” “seafood”), and omega-3 fatty acids (“fish oil,” “omega-3,” “n-3 fatty acids,” “n-3 fatty acid”) (27). No language restrictions were set in the search strategy. We first screened the titles and abstracts of all publications. Then, the full articles were read to identify eligible studies. To avoid missing literature, a manual search was also conducted from the reference lists of all articles selected for inclusion.

### Study Selection

The title and abstract screening of relevant articles was done separately by two researchers (YZ and JD) to identify eligible studies for inclusion. The potentially eligible articles were selected through full text review in line with the inclusion and exclusion criteria according to PICOS strategy. The included studies were required to meet the following criteria: (1) the participants were general population; (2) the exposure of interest was the consumption of fish or omega-3 fatty acids; (3) the comparison was the highest vs. lowest category of exposure; (4) the outcomes included the risk of VTE; (5) prospective cohort studies. The exclusion criteria were listed as follows: (1) duplicated or irrelevant articles; (2) reviews, letters or case reports; (3) randomized controlled trials; (4) non-human studies.

### Data Extraction

Data extraction was conducted by two independent reviewers (YZ and JD); disagreements were resolved by consensus. The following information was collected: first author, year of publication, location, age, gender, sample size, number of cases, follow-up, outcome, category of exposure, effect estimates, and adjustments. The corresponding effect estimates adjusted for the maximum number of confounding variables with corresponding 95% CIs for the highest vs. lowest level were extracted. For the studies reported effect estimates by gender separately, they were processed independently (22). Moreover, Varraso' study was consisted of two different cohorts: the NHS (Nurses' Health Study) and HPFS (Health Professionals Follow-up Study) (21). They were considered as two independent studies.

### Quality Assessment

Quality assessment was conducted according to the Newcastle-Ottawa (NOS) criteria for non-randomized studies (28), which is based on three broad perspectives: the selection process of study cohorts, the comparability among different cohorts, and the identification of either the exposure or outcome of study cohorts. Disagreements with respect to the methodological quality were resolved by discussion and mutual-consultation.

### Statistical Analyses

RR was considered as the common measure of the associations of fish and omega-3 fatty acids consumption with the risk of VTE, and HR was directly converted into RR. The *I*^2^ statistic, which measures the percentage of the total variation across studies due to heterogeneity, was examined (*I*^2^ > 50% was considered heterogeneity). If significant heterogeneity was observed among studies, the random-effects model was used; otherwise, the fixed-effects model was acceptable. Begg's tests were performed to assess the publication bias (29), and statistical analyses were performed using STATA version 11.0 (StataCorp LP, College Station, Texas). A *p*-value ≤ 0.05 was accepted as statistically significant. Subgroup analysis for sample size, gender, location and adjustment of cigarette smoking, physical activity and hypertension, were conducted.

## Results

### Study Identification and Selection

**Figure 1:**
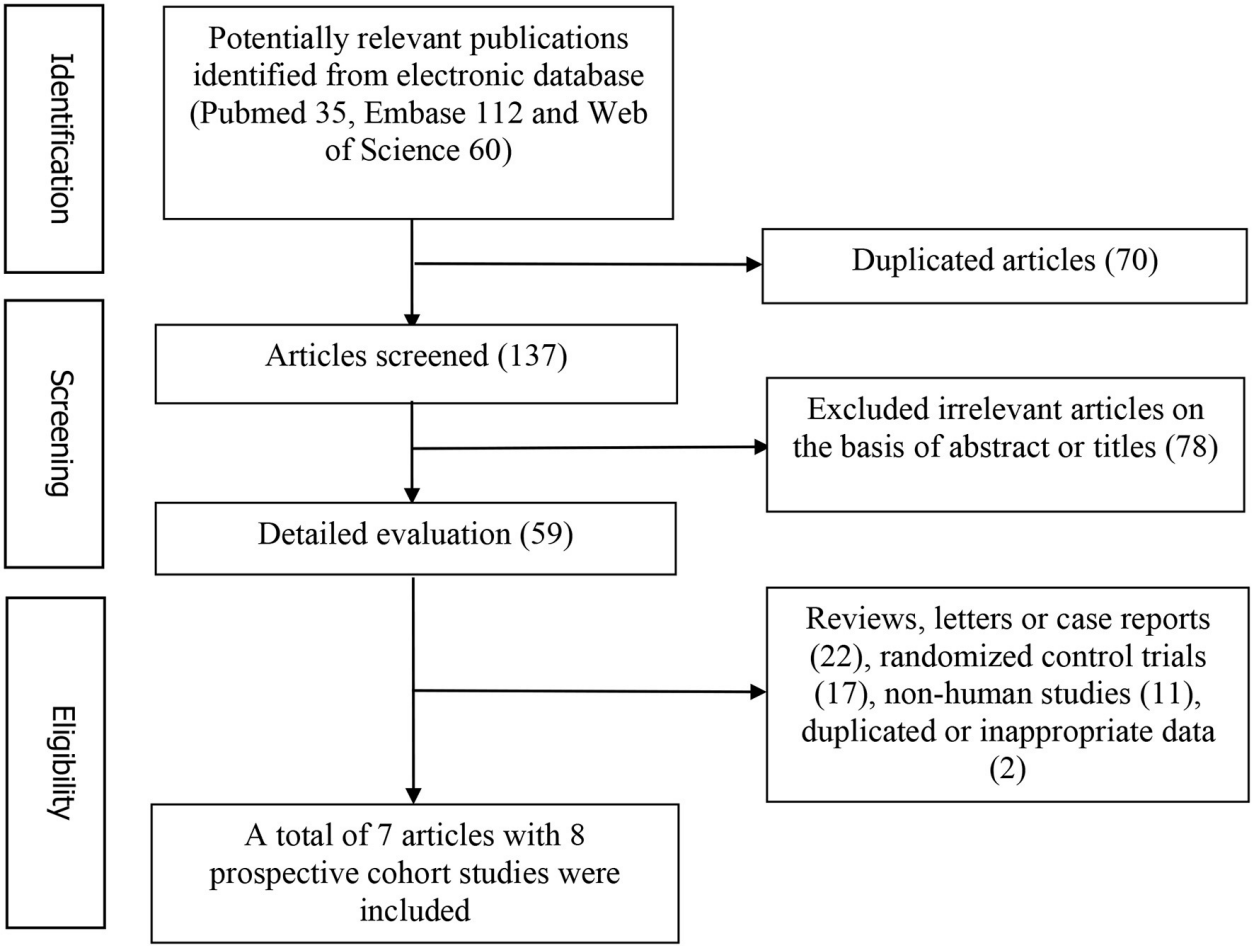
Flow chart for the identification of studies that were included in this meta-analysis.

Figure 1 represents the detailed flow diagram of articles included in the present meta-analysis. Our initial literature searches yielded a total of 207 potentially relevant articles (PubMed 35, Embase 112, and Web of Science 60). After eliminating 70 duplicated articles, 137 articles were screened by titles and abstracts. 78 irrelevant studies, 22 reviews, case reports or letters, 11 non-human studies, 17 randomized control trials studies and two studies with duplicated or inappropriate data (outcome was PE death) were removed (30, 31). Eventually, a total of seven articles with eight prospective studies were identified for this meta-analysis (13, 19–24).

### Study Characteristics

The main characteristics of the included studies were showed in Table 1. These studies were published between 2006 and 2019, which included eight prospective cohort studies. Four studies were performed in USA (19–21) and the other four ones were conducted in European country [Norway (13, 23), Denmark (22), Switzerland (24)]. The follow-up duration ranged from 0.5 to 19 years. Five studies included both male and female participants (13, 19, 22–24), and three studies included only female or male participants (20, 21). The sample size ranged from 595 to 80,263. The fish and omega-3 fatty acids consumption was assessed by food-frequency questionnaire (FFQ) in all studies. The diagnosis of VTE was obtained in registered medical record (imaging or autopsy) and food was considered as the source of omega-3 fatty acids in all included studies. Six and four studies were related to the associations of fish (19–23) and omega-3 fatty acids (19, 21, 23) consumption with the risk of VTE, respectively. Two studies were related to the relationship between omega-3 fatty acids consumption and the risk of recurrent VTE (13, 24).

**Table 1:** Characteristics of the individual studies included in this meta-analysis.

**References**	**Location**	**Age and BMI**	**Gender**	**Sample Size**	**Number of cases**	**Follow-up years**	**Outcome**	**Category of exposure**	**Effect estimates**	**Adjustments**	**NOS**
Steffen (19)	USA	54 27.5	Both	14,962	196	12.5	VTE	Fish Quintile 1 Quintile 2 Quintile 3 Quintile 4 Quintile 5 Omega-3 Fatty Acids Quintile 1 Quintile 2 Quintile 3 Quintile 4 Quintile 5	1 0.58 (0.37, 0.90) 0.60 (0.39, 0.92) 0.55 (0.35, 0.88) 0.70 (0.44, 1.10) 1 0.56 (0.36, 0.87) 0.64 (0.42, 0.99) 0.54 (0.34, 0.85) 0.70 (0.43, 1.13)	Age, race, gender, field center, energy intake, vitamin supplement use, BMI, diabetes, vegetable, fruit, whole grain, red and processed meat	8
Lutsey (20)	USA	55–69 27	Female	37,393	1,950	19	VTE	Fish <0.5 Servings/week 0.5-1 Servings/week 1-1.5 Servings/week 1.5-2.5 Servings/week ≥2.5 Servings/week	1 1.13 (0.95, 1.34) 1.16 (0.98, 1.36) 1.07 (0.91, 1.26) 1.22 (1.03, 1.46)	Age, energy intake, education, smoking status, and physical activity, BMI, diabetes	7
Varraso NHS (21)	USA	30–55 25	Female	80,263	1,540	14	VTE	Fish Quintile 1 Quintile 2 Quintile 3 Quintile 4 Quintile 5 Omega-3 Fatty Acids Quintile 1 Quintile 2 Quintile 3 Quintile 4 Quintile 5	1 0.94 (0.80, 1.11) 0.92 (0.79, 1.09) 0.96 (0.82, 1.13) 0.95 (0.80, 1.11) 1 0.96 (0.82, 1.12) 0.92 (0.78, 1.08) 0.98 (0.84, 1.15) 0.94 (0.80, 1.10)	Age, total physical activity level, physical inactivity level, BMI, total caloric intake, smoking, pack-years of smoking, race/ethnicity, spouse's educational attainment, parity, menopausal status, nonaspirin nonsteroidal anti-inflammatory drug use, warfarin use, multivitamin supplement use, hypertension, coronary heart disease, and rheumatologic disease	9
Varraso HPFS (21)	USA	40–75 25	Male	49,238	1,352	12	VTE	Fish Quintile 1 Quintile 2 Quintile 3 Quintile 4 Quintile 5 Omega-3 Fatty Acids Quintile 1 Quintile 2 Quintile 3 Quintile 4 Quintile 5	1 1.02 (0.85, 1.22) 0.83 (0.69, 0.99) 0.96 (0.81, 1.15) 0.96 (0.80, 1.14) 1 1.01 (0.86, 1.19) 0.88 (0.74, 1.04) 0.91 (0.77, 1.08) 0.91 (0.76, 1.08)	Age, total physical activity level, physical inactivity level, BMI, total caloric intake, smoking, pack-years of smoking, race/ethnicity, spouse's educational attainment, parity, menopausal status, nonaspirin nonsteroidal anti-inflammatory drug use, warfarin use, multivitamin supplement use, hypertension, coronary heart disease, and rheumatologic disease	9
Varraso HPFS (21)	USA	40–75 25	Male	49,238	1,352	12	VTE	Fish Quintile 1 Quintile 2 Quintile 3 Quintile 4 Quintile 5 Omega-3 Fatty Acids Quintile 1 Quintile 2 Quintile 3 Quintile 14 Quintile 5	1 1.02 (0.85, 1.22) 0.83 (0.69, 0.99) 0.96 (0.81, 1.15) 0.96 (0.80, 1.14) 1 1.01 (0.86, 1.19) 0.88 (0.74, 1.04) 0.91 (0.77, 1.08) 0.91 (0.76, 1.08)	Age, total physical activity level, physical inactivity level, BMI, total caloric intake, smoking, pack-years of smoking, race/ethnicity, spouse's educational attainment, parity, menopausal status, nonaspirin nonsteroidal anti-inflammatory drug use, warfarin use, multivitamin supplement use, hypertension, coronary heart disease, and rheumatologic disease	9
Severinsen Male (22)	Denmark	50–64 26.2	Male	26,674	641	10.2	VTE	Fish Quintile 1 Quintile 2 Quintile 3 Quintile 4 Quintile 5	1 0.98 (0.71, 1.35) 1.09 (0.78, 1.51) 1.02 (0.74, 1.42) 0.90 (0.64, 1.28)	Total energy intake, smoking, BMI, dietary intake of fruits and vegetables	7
Severinsen Female (22)	Denmark	50–64 24.8	Female	29,340	641	10.2	VTE	Fish Quintile 1 Quintile 2 Quintile 3 Quintile 4 Quintile 5	1 0.70 (0.47, 1.04) 0.74 (0.50, 1.10) 0.85 (0.57, 1.27) 1.19 (0.77, 1.83)	Total energy intake, smoking, BMI, use of hormone replacement therapy, dietary intake of fruits and vegetables	7
Reiner (24)	Switzerland	75 26.4	Both	826	97	0.5	Recurrent VTE	Omega-3 Fatty Acids Low Medium High	1 0.39 (0.15, 0.99) 0.17 (0.03, 0.96)	Age, gender, BMI, cancer, provoked VTE, prior VTE and periods of anticoagulation as a time-varying covariate	7
Isaksen (23)	Norway	25–97 25.5	Both	21,970	541	12.5	VTE	Fish Quartile 1 Quartile 2 Quartile 3 Quartile 4 Omega-3 Fatty Acids Quartile 1 Quartile 2 Quartile 3 Quartile 4	1 0.93 (0.71, 1.23) 0.98 (0.75, 1.27) 0.99 (0.76, 1.29) 1 0.74 (0.57, 0.96) 0.77 (0.59, 0.99) 0.78 (0.61, 1.00)	Age, sex and BMI	8
Isaksen (13)	Norway	25–97 27.4	Both	595	98	12.5	Recurrent VTE	Omega-3 Fatty Acids Quartile 1 Quartile 2 Quartile 3	1 1.01 (0.61, 1.66) 0.51 (0.27, 0.95)	Age, sex, and BMI	7

### Association Between Fish Consumption and the Risk of VTE

**Figure 2:**
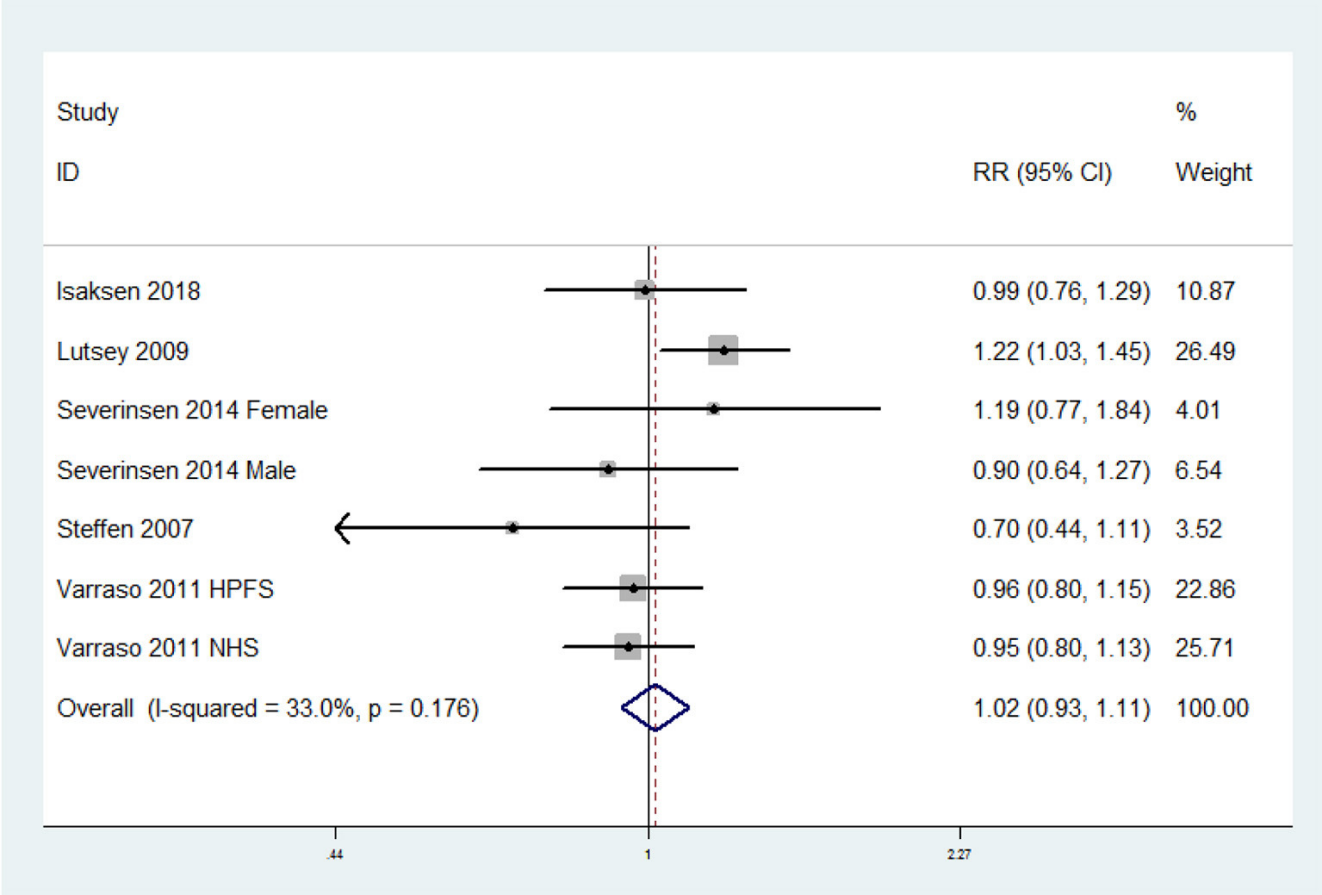
Forest plot of meta-analysis: Overall multi-variable adjusted RR of VTE for the highest vs. lowest category of fish consumption.

The overall multi-variable adjusted RR suggested no significant relationship between fish consumption and the risk of VTE (RR = 1.02, 95% CI: 0.93–1.11; *P* = 0.709) (Figure 2). No substantial level of heterogeneity was found among various studies (*P* = 0.176, *I*^2^ = 33%). Begg's rank-correlation test showed no evidence of publication bias (*P* = 0.548). The results of subgroup analysis were showed in Table 2. The sensitivity analysis showed only minimal changes in magnitude of the pooled RR when any study was excluded from the meta-analysis, suggesting that no individual study had excessive influence on these robust aggregate results (data not shown).

**Table 2:** Subgroup analyses of fish consumption and the risk of VTE.

**Subgroup**	**Number of studies**	**Pooled RR**	**95% CI**	**Heterogeneity**
All**Sample size**	6	1.02	0.93–1.11	*P* = 0.18; *I*^2^ = 33%
<30,000	2	0.91	0.72–1.14	*P* = 0.20; *I*^2^ = 38%
>30,000	4	1.04	0.94–1.14	*P* = 0.18; *I*^2^ = 36%
**Gender**				
Male	2	0.95	0.81–1.11	*P* = 0.74; *I*^2^ = 0%
Female	3	1.09	0.90–1.32	*P* = 0.12; *I*^2^ = 54%
**Location**				
USA	4	1.00	0.84–1.18	*P* = 0.05; *I*^2^ = 62%
Other	2	1.00	0.82–1.20	*P* = 0.61; *I*^2^ =0%
**Adjustment of cigarette smoking**				
Adjusted	4	1.04	0.94–1.14	*P* = 0.18; *I*^2^ = 36%
Unadjusted	2	0.91	0.72–1.14	*P* = 0.20; *I*^2^ = 38%
**Adjustment of physical activity**				
Adjusted	3	1.04	0.88–1.22	*P* = 0.07; *I*^2^ = 62%
Unadjusted	3	0.95	0.80–1.13	*P* = 0.41; *I*^2^ = 0%
**Adjustment of hypertension**				
Adjusted	2	0.95	0.84–1.08	*P* = 0.93; *I*^2^ = 0%
Unadjusted	4	1.08	0.96–1.22	*P* = 0.13; *I*^2^ = 43%

### Association Between Omega-3 Fatty Acids Consumption and the Risk of VTE

**Figure 3:**
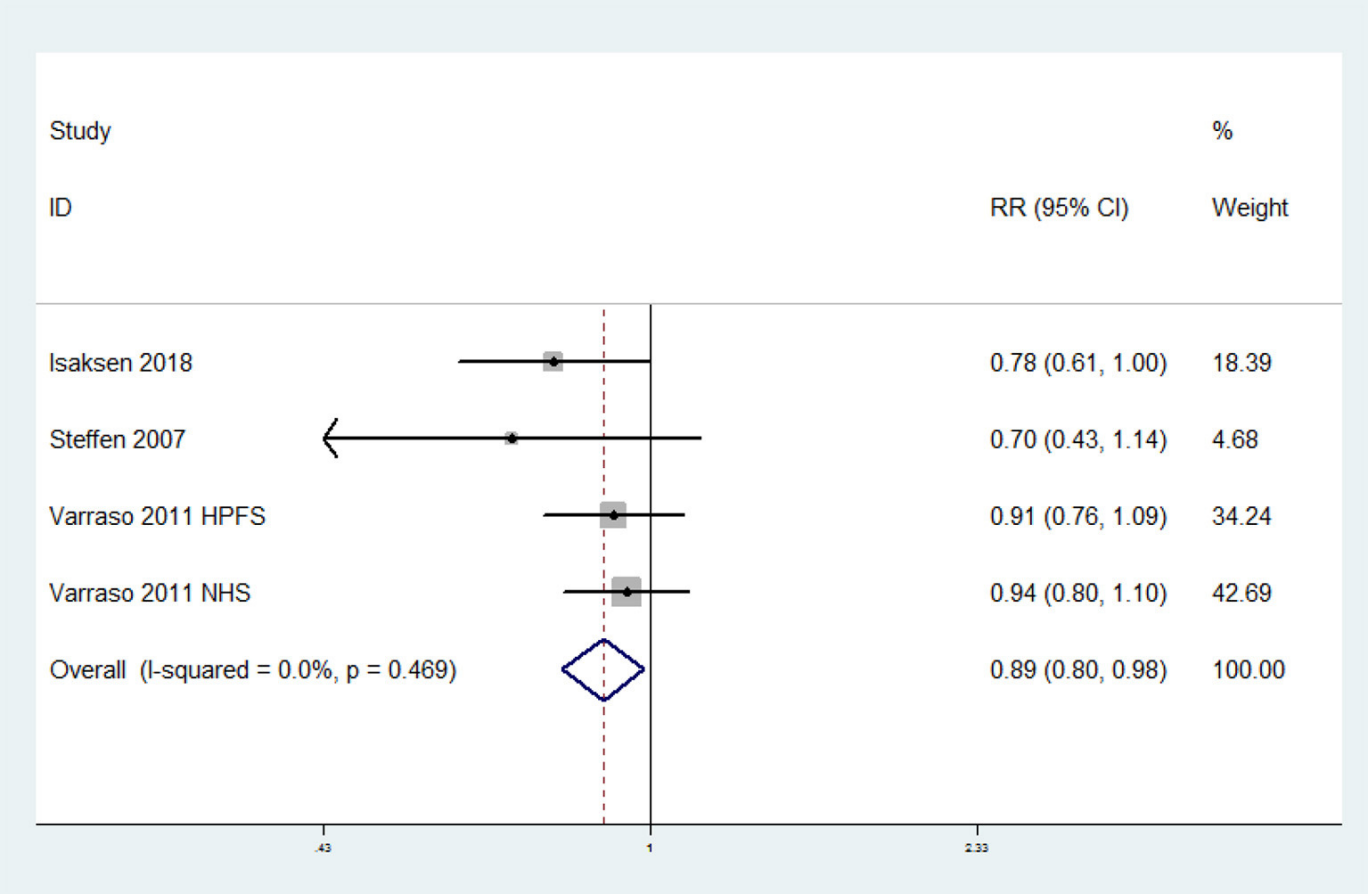
Forest plot of meta-analysis: Overall multi-variable adjusted RR of VTE for the highest vs. lowest category of omega-3 fatty acids consumption.

The overall multi-variable adjusted RR suggested that omega-3 fatty acids consumption was associated with a lower risk of VTE (RR = 0.89, 95% CI: 0.80–0.98; *P* = 0.024) (Figure 3). No substantial level of heterogeneity was found among various studies (*P* = 0.469, *I*^2^ = 0%). Begg's rank-correlation test showed no evidence of publication bias (*P* = 0.308). The results of subgroup analysis were showed in Table 3. The sensitivity analysis showed only minimal changes in magnitude of the pooled RR when any study was excluded from the meta-analysis, suggesting that no individual study had excessive influence on these robust aggregate results (data not shown).

**Table 3:** Subgroup analyses of omega-3 fatty acids consumption and the risk of VTE.

**Subgroup**	**Number of studies**	**Pooled RR**	**95% CI**	**Heterogeneity**
All**Sample size**	4	0.89	0.80–0.98	*P* = 0.47; *I*^2^ = 0%
<30,000	2	0.76	0.61–0.95	*P* = 0.70; *I*^2^ = 0%
>30,000	2	0.93	0.82–1.04	*P* = 0.79; *I*^2^ = 0%
**Gender**				
Male	1	0.91	0.76–1.09	/
Female	1	0.94	0.80–1.10	/
**Location**				
USA	3	0.91	0.81–1.02	*P* = 0.53; *I*^2^ = 0%
Other	1	0.78	0.61–1.00	/
**Adjustment of cigarette smoking**				
Adjusted	2	0.93	0.82–1.04	*P* = 0.79; *I*^2^ = 0%
Unadjusted	2	0.76	0.61–0.95	*P* = 0.70; *I*^2^ = 0%
**Adjustment of physical activity**				
Adjusted	2	0.93	0.82–1.04	*P* = 0.79; *I*^2^ = 0%
Unadjusted	2	0.76	0.61–0.95	*P* = 0.70; *I*^2^ = 0%
**Adjustment of hypertension**				
Adjusted	2	0.93	0.82–1.04	*P* = 0.79; *I*^2^ = 0%
Unadjusted	2	0.76	0.61–0.95	*P* = 0.70; *I*^2^ = 0%

### Association Between Omega-3 Fatty Acids Consumption and the Risk of Recurrent VTE

**Figure 4:**
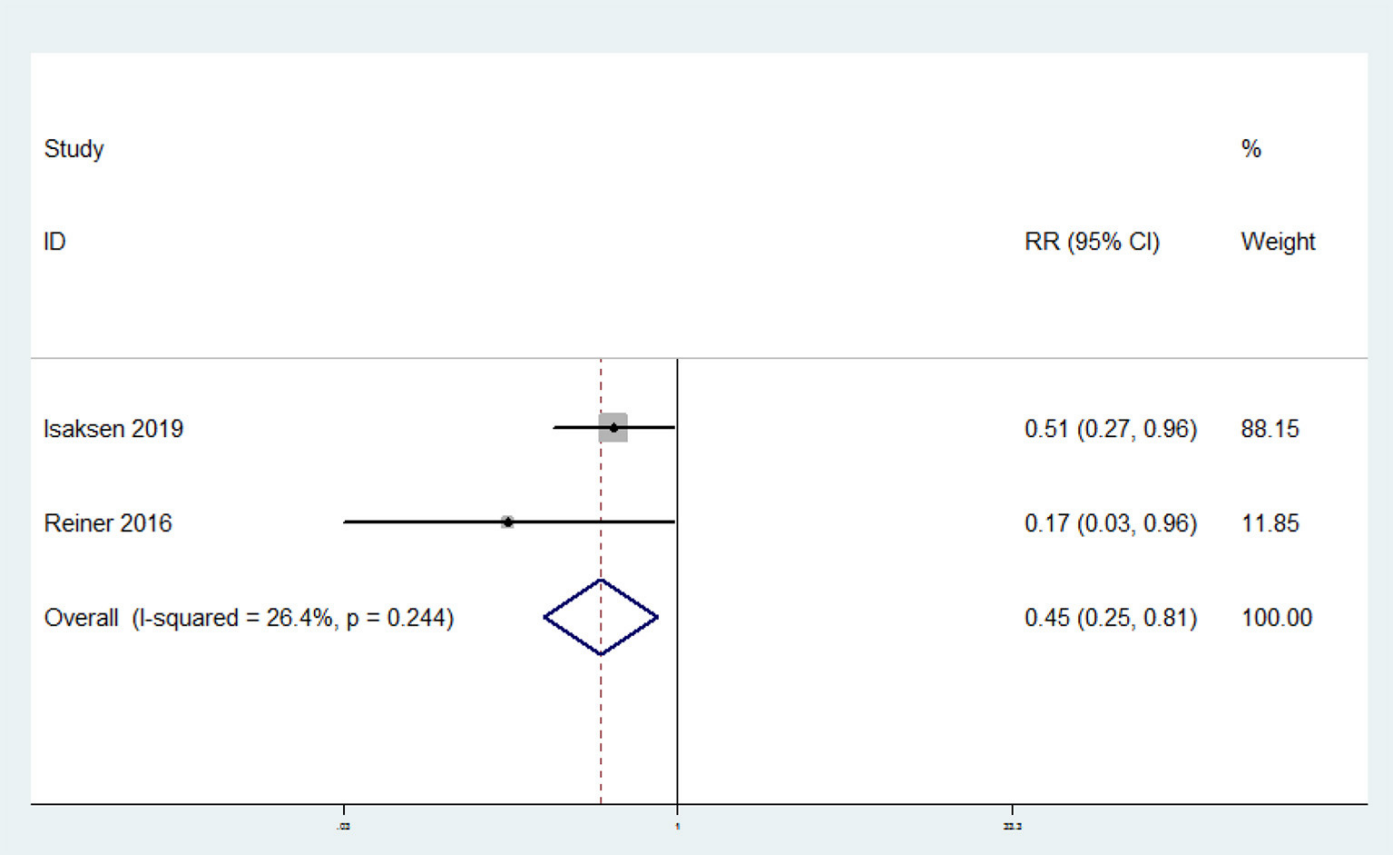
Forest plot of meta-analysis: Overall multi-variable adjusted RR of recurrent VTE for the highest vs. lowest category of omega-3 fatty acids consumption.

The overall multi-variable adjusted RR showed that omega-3 fatty acids consumption was associated with a lower risk of recurrent VTE (RR = 0.45, 95% CI: 0.25–0.81; *P* = 0.008) (Figure 4). No substantial level of heterogeneity was found among various studies (*P* = 0.244, *I*^2^ = 26.4%). Begg's rank-correlation test showed no evidence of publication bias (*P* = 1.00).

## Discussions

In the present meta-analysis, a total of eight prospective cohort studies were identified for examination. No significant relationship between fish consumption and the risk of VTE was obtained, whereas omega-3 fatty acids consumption was associated with a lower risk of both VTE and recurrent VTE.

Several studies have demonstrated the impact from omega-3 fatty acids on downregulation of inflammation (14), tissue factor expression (18, 32), platelet function (15), platelet-endothelium interactions (16, 17), and hepatic excretion of coagulation factors (33), which are significant molecular pathways in VTE pathogenesis. Omega-3 fatty acids may also alternate cell membrane permeability and functionality, which leads to antiarrhythmic properties (14). Moreover, some randomized control trials demonstrated that omega-3 fatty acids (both supplementation and circulating level) was associated with a lower risk of VTE after orthopedics surgery (34, 35). On the other hand, a cross-sectional observational study also found a negative relationship between fish consumption and VTE (36). These findings strongly support the potential beneficial effect of omega-3 fatty acids. However, inconsistent results were obtained with regard to experimental animal study. One study demonstrated that omega-3 fatty acids could prevent VTE (37), while some other studies rejected it (38, 39). These inconsistent results in rodents may be difficult to translate to human, especially elderly patients with various risk factors. Moreover, the differences in VTE pathophysiology may account for this discrepancy. The low-dose and long-term protective mechanisms of omega-3 fatty acids may apply to elderly patients with chronic pro-inflammatory and pro-thrombotic backgrounds, whereas animal models consist of an acute and short-term injury with a relatively short exposure to omega-3 fatty acids (24). Therefore, further animal study will be helpful to deepen our understanding.

Notably, this topic has been discussed by a systematic review in 2016 by Mattiuzzi et al. (25). It claimed that the epidemiological evidence was insufficient to demonstrate any relationship between fish consumption and the risk of VTE. However, the estimate effects were not pooled and omega-3 fatty acids and recurrent VTE were also ignored. It also indicated that cigarette smoking and physical activity should be considered as confounders. To address these issues above, the present study was therefore systematically preformed. We found an inverse association between omega-3 fatty acids consumption and the risk of VTE, which disappeared when cigarette smoking, physical activity and hypertension was adjusted (Table 3). Nevertheless, the reliability of results may be reduced by the small number of studies. As a consequence, more studies with adjustment of these confounding factors are still needed.

So far, the bioavailability of the omega-3 fatty acids is rarely considered in defeating diseases. The formation of phospholipids and regulation of membrane properties are closely associated with the biological effect of omega-3 fatty acids. For example, the omega-3 fatty acids content in erythrocyte membranes (40) and fatty acid-based membrane lipidomics (41) are served as important reliable indicator for omega-3 fatty acids consumption. On the contrary, as the most general indicator, FFQ could not reflect its biological effective quantity in the body. Further studies with bioavailability of omega-3 fatty acids are still needed. On the other hand, as another important polyunsaturated fatty acid, omega-6 fatty acids should also be considered. The metabolites of omega-6 fatty acids are proinflammatory/proaggregatory agent (42), which contributes to the risk of all-cause mortality, coronary heart disease mortality and cardiovascular events (43). Indeed, omega-3 and omega-6 fatty acids are supposed to balance each other when they are consumed in the diet at a ratio of around 1 to 1 (44). The increase in omega-6/omega-3 fatty acids ratio could shift the balance into a proinflammatory/proaggregatory state, and contributed to platelet aggregation, coagulation and thrombosis (44). Therefore, more studies with detail information on omega-6 fatty acids consumption are needed.

Interestingly, the associations of fish and omega-3 fatty acids consumption with the risk of VTE are inconsistence. Several speculations may account for this discrepancy. First, the reliability of the results might be weakened since only limited studies were included. Second, some neglected substances might work against the effect of omega-3 fatty acids since the components in fish are rather complicated. Third, the processing method of fish may also matter. The effect from fresh fish consumption on VTE risk was stronger than that from dried or salted fish, steamed fish paste and deep-fried fish (omega-3 fatty acids were mainly derived from fresh fish) (31). Fourth, the content of omega-3 fatty acids in fatty fish can be up to 7–8 folds higher than that in lean fish. It is possible that a high intake of (lean) fish is associated with a relatively low intake of omega-3 fatty acids (23). On the other hand, different results were obtained with regard to the category of VTE (provoked and unprovoked, DVT and PE) (13, 23). However, the limitation of current evidence precluded a subgroup analysis. More well-designed prospective cohort studies with fish and VTE specification are needed.

Our study has several strengths. First, this is the first meta-analysis of prospective cohort studies aiming at the associations of fish and omega-3 fatty acids consumption with the risk of VTE based on the most comprehensive literature search to date. Second, most of the included studies were analyzed based on adjusted results and large samples. Third, our result may be helpful for health professionals and policy makers to better consider the effect of diet on VTE risk. However, this meta-analysis was also limited in some aspects. First, due to the limitation of relevant literature, only 8 prospective cohort studies were identified. Second, the classification of exposure may vary greatly among individuals. Third, the selection of adjusted factors was not uniform. Fourth, only a few studies have considered the processing method of fish and category of fish (lean and fatty) or VTE (provoked and unprovoked, DVT and PE), some issues could not be addressed. Last but not the least, the consumption of omega-6 fatty acids, which could influence the results of our study, was not considered. These limitations might weaken the significance of this study.

## Conclusions

Although current evidence is still insufficient to demonstrate any relationship between fish consumption and the risk of VTE, omega-3 fatty acids consumption seems to be associated with a lower risk of both VTE and recurrent VTE. However, further well-designed prospective cohort studies are still needed.

## Data Availability Statement

The original contributions presented in the study are included in the article/Supplementary Material, further inquiries can be directed to the corresponding author/s.

## Author Contributions

YZ conceived the idea, performed the statistical analysis, and drafted this meta-analysis. YZ and JD selected retrieved relevant papers. HG assessed each study. JL and YL were the guarantors of the overall content. All authors revised and approved the final manuscript.

## Conflict of Interest

The authors declare that the research was conducted in the absence of any commercial or financial relationships that could be construed as a potential conflict of interest.
