# Supplementary material for: Associations of Fish and Omega-3 Fatty Acids Consumption With the Risk of Venous Thromboembolism. A Meta-Analysis of Prospective Cohort Studies
Source: Front Nutr. 2020 Dec 17;7:614784. doi: 10.3389/fnut.2020.614784 (PMC7793732; doi:10.3389/fnut.2020.614784)
Supplement: Supplementary file 1 [file Table_1.DOCX]

**Search Strategy**

(((fish[Title/Abstract]) OR (seafood[Title/Abstract])) OR (((fish oil[Title/Abstract]) OR (Omega-3[Title/Abstract])) OR (n-3 Fatty Acid*[Title/Abstract]))) AND (((venous thromboembolism[Title/Abstract]) OR (deep vein thrombosis[Title/Abstract])) OR (pulmonary embolism[Title/Abstract]))
